# Baseline Testosterone Levels Peak During the Inactive Period in Male Degus (*Octodon degus*)

**DOI:** 10.1093/iob/obaf033

**Published:** 2025-07-29

**Authors:** Y Sato, T M S Garcia, J S Low, C M Bauer

**Affiliations:** Department of Biology, Swarthmore College, Swarthmore, PA 19081, USA; Department of Biology, Swarthmore College, Swarthmore, PA 19081, USA; Department of Biology, Swarthmore College, Swarthmore, PA 19081, USA; Department of Biology, Swarthmore College, Swarthmore, PA 19081, USA

## Abstract

The steroid hormone testosterone is important for stimulating male reproductive processes including territory acquisition, mating displays, and spermatogenesis. When examining the relative effects of testosterone on reproductive processes, it is most reasonable to focus on peak baseline testosterone levels, especially for reproductive processes that may occur during specific times of day, such as mating and spermatogenesis. However, some studies have not found consistent positive relationships between circulating testosterone levels and reproductive variables. These nonsignificant relationships could be driven by methodology, as most studies in wild, free-living animals collect blood samples during an animals' active period, yet many species show peak baseline testosterone levels during their inactive period. This may be the case for the common degu (*Octodon degus*), as field and laboratory studies have exclusively sampled these diurnal rodents during their active period and have found little correlation between testosterone levels and reproductive success. In this study, we measured testosterone levels in captive male degus every 4 h across a 24-h cycle to test the hypothesis that male degus demonstrate diel variation in their baseline testosterone levels. We saw significant variation in male degu baseline testosterone levels over a 24-h period, and our prediction that baseline testosterone levels would be higher during nighttime (inactive period) timepoints compared to daytime (active period) timepoints was supported. However, nighttime baseline testosterone levels were still several magnitudes lower than testosterone levels after a gonadotropin-releasing hormone (GnRH) injection. While GnRH injections significantly increased circulating plasma testosterone levels during any daytime time period, we found no significant correlation between nighttime baseline testosterone levels and post-GnRH testosterone levels, which suggests GnRH-challenges during the daytime cannot be used to approximate or estimate nighttime baseline testosterone levels. These findings expand our knowledge surrounding testosterone dynamics and suggest that future studies should take into account the time of day when sampling testosterone and other hormone levels.

## Introduction

Testosterone is a steroid hormone that supports reproductive behaviors and physiological processes in vertebrates (Hau 2007). Testosterone is ultimately secreted upon activation of the hypothalamic-pituitary-gonadal (HPG) axis, whereupon release of endogenous gonadotropin-releasing hormone (GnRH) from the hypothalamus activates secretion of luteinizing hormone from the pituitary gland, which then travels in the bloodstream and stimulates production and secretion of reproductive steroid hormones, such as testosterone, from the gonads. While testosterone promotes important reproductive functions in both males and females, testosterone is best known for its many functions in males including enhancing territoriality, facilitating mating displays, and stimulating spermatogenesis (Zielinski and Vandenbergh 1993; Blottner et al. 2000). Circulating plasma levels of testosterone often show seasonal rhythms (Hau 2007), usually peaking during the breeding season (e.g., the coyote [*Canis latrans*]; [Minter and DeLiberto 2008], and the striped mouse [*Rhabdomys pumilio*]; [Schradin 2008]).

However, links between testosterone and reproductive parameters are not always straightforward, and depend on several different factors including social context (Kelly and Thompson 2023), age (Martin et al. 2013), past experience (Oyegbile and Marler 2005), and environmental conditions (Gesquiere et al. 2011). While many past studies have demonstrated positive correlations between circulating testosterone levels and different reproductive variables in male vertebrates, other studies fail to find these relationships (Book et al. 2001; Sannen et al. 2004; Correa et al. 2024b; Apfelbeck et al. 2013; Hau and Goymann 2015). One reason why some studies may fail to find these positive relationships could be due to the timing of sample collection, as researchers often collect samples during a species' active period. However, testosterone shows strong diel rhythms in vertebrates, with peaks generally occurring during the inactive period. Diurnal mammalian species such as the northern palm squirrel (*Funambulus pennantii)* and rhesus macaque (*Macaca mulatta*) display peaks in testosterone during the nighttime (Perachio et al. 1977; Haldar et al. 2006). Similarly, nocturnal mammalian species such as mouse lemurs (*Microcebus murinus*; [Perret 1985]), owl monkeys (*Aotus trivirgatus*; [Dixson and Gardner 1981]), and domestic mice (*Mus musculus*; [Li et al. 2020]) display testosterone peaks during the daytime. Peak levels of testosterone may be more indicative of reproductive success (McGlothlin et al. 2007), likely because more individual variation can be detected during these timepoints. As wild animals are generally sampled during their active period, typical sampling paradigms may be missing important relationships.

Degus (*Octodon degus*) are one such species where lack of relationships between male testosterone levels and reproductive fitness are likely due to nonideal sampling periods. Degus are small, diurnal caviomorph rodents native to central Chile, and a past study found that wild, free-living male degus display higher circulating plasma levels of testosterone during the mating season compared to other times of year (Soto-Gamboa 2005), and that male-male agonistic encounters are also more frequent during this time period. However, other studies have found that male degu testosterone levels are highest during the offspring rearing season, and that male testosterone levels do not significantly relate with the number of female mating partners nor with the number of sired offspring (Correa et al. 2024a, 2024b). Samples from these studies were collected during the degus' active period, which may not reflect peak baseline testosterone levels and therefore may be obscuring significant correlations between testosterone and different reproductive variables.

While collecting blood samples during the inactive period may improve our resolution to detect correlations between testosterone levels and reproductive parameters, it should be noted that pharmacological techniques can be used to induce peak testosterone levels during the active period. For example, injections of GnRH can increase testosterone levels via stimulation of the HPG-axis (Kriegsfeld et al. 1999; Jawor et al. 2006; Matas et al. 2020). These GnRH challenges can assess peak testosterone levels, with the idea that a high dose of GnRH may stimulate maximal, potential levels of testosterone in males. Past studies have found that GnRH-induced testosterone can be a strong predictor of reproductive success in a wild songbird species (McGlothlin et al. 2007, 2010). Additional songbird studies have also found that GnRH-induced testosterone levels are tightly correlated with peak baseline testosterone levels (Needham et al. 2017; Greives et al. 2021), although no mammal studies to date have looked at this relationship. Therefore, GnRH challenges may provide a reliable proxy of peak baseline testosterone levels in mammalian species while still allowing animals to be sampled during their active period.

In this study, we tested the hypothesis that male degus display peak baseline testosterone levels during their inactive period. To test this hypothesis, we measured plasma testosterone levels in a cohort of similarly aged captive male degus at several time points during their active and inactive periods: 2, 6, and 10 h after lights are turned on in the morning and 2, 6, and 10 h after lights turned off in the evening. We also performed GnRH challenges immediately after collecting baseline testosterone samples during the active period. We predicted that if male degu baseline testosterone levels peak during their inactive period, then testosterone levels would be higher during the lights-off time points compared to the lights-on time points. We predicted that if daytime GnRH-induced testosterone levels are a good proxy of peak baseline testosterone levels, then there should be a positive correlation between these variables within our male degu cohort.

## Methods

### Animal husbandry

We used reproductively active 3-year-old male degus (*n* = 18) from a laboratory colony at Swarthmore College. Males were singly housed at 20°C in 500 × 230 × 320 mm acrylic terrariums and provided with *ad libitum* food (5001 Laboratory Rodent Diet, LabDiet, St. Louis, MO) and water. Degus experienced a 12L:12D light cycle, with lights on at 08:00 and off at 20:00; prior studies have found that degus readily mate under this photoperiod (Palacios and Lee 2013). Each cage contained corn cob bedding, manzanita chew sticks, a free-standing wire wheel, and supplemental timothy hay.

### Blood sampling and injections

**Fig. 1.**
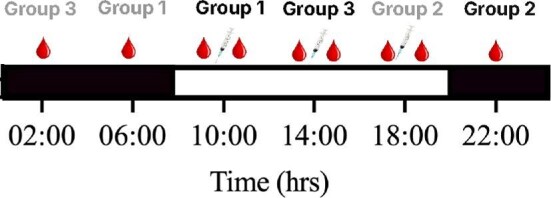
Experimental timeline of sampling reproductively active, singly housed, 3-year-old male degus (*Octodon degus*) for testosterone. Black and white bars indicate the times of day when lights were off and on, respectively. Degus were split into three groups (*n* = 6 males per group). Black and gray text indicate a group's first and second sampling timepoint, respectively; each group received 2 weeks of recovery in between their sampling timepoints. During lights-off sampling time points, only baseline testosterone samples were taken (one blood droplet), whereas during lights-on time points, testosterone was measured at both baseline and after injection of gonadotropin-releasing hormone (two blood droplets surrounding a syringe).

Adult male degus were split into three groups (*n* = 6) and each group was sampled once during lights-on (10:00, 14:00, or 18:00) and once during lights-off (22:00, 02:00, or 06:00) (Fig. 1). All degus within each group were sampled for the same lights-on and lights-off periods (22:00 and 18:00, 14:00 and 02:00, or 10:00 and 06:00), and we waited at least 2 weeks between sampling time points for each group. The facility was left undisturbed for at least 1 h before each sampling period, and each timepoint was sampled on a different day.

**Fig. 2.**
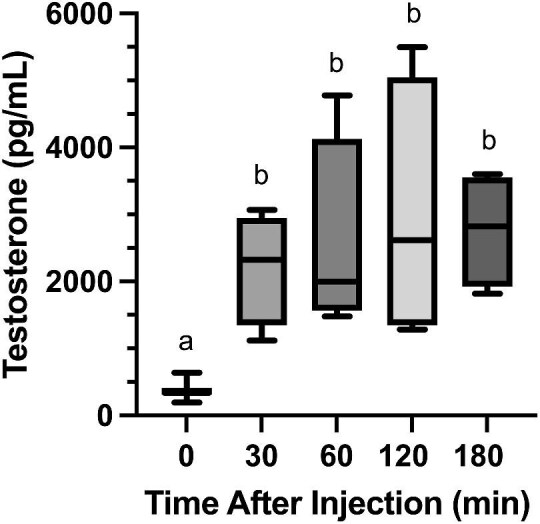
Plasma testosterone levels in male degus 0 (*n* = 16), 30 (*n* = 4), 60 (*n* = 4), 120 (*n* = 4), and 180 (*n* = 4) min after injection of gonadotropin-releasing hormone. Boxes represent 25th–75th percentiles, middle lines represent medians, and whiskers represent minimum and maximum values. Different letters indicate significant differences between timepoints.

To collect blood samples, we pricked the saphenous vein using 25G needles and collected blood (210–280 µL for baseline samples, 140–210 µL for post-GnRH samples) using heparinized microhematocrit capillary tubes. Samples were collected within 30 min of entering the animal facility and placed on ice for up to 2 h before centrifugation. For daytime sampling periods (10:00, 14:00, or 18:00), immediately after collecting baseline blood samples, we injected degus with a 2 mg/kg of body weight dose (Greives et al. 2021) of gonadotropin-releasing hormone (LHRH acetate salt, product # 4033013, Bachem, Torrance, CA) into the peritoneal cavity and collected a blood sample 2 h after injection. We confirmed in a preliminary study that maximal testosterone levels occurred 2 h after GnRH injections in degus, as testosterone levels significantly increased 30 min after GnRH injection (Fig. 2: ANOVA: F_4,27_ = 12.33, *P* < 0.001, post-hoc LSD t_0_ vs. t_30_  *P* = 0.001) and remained unchanged for the next 2.5 h (post-hoc LSDs t_30_ vs. t_60_, t_120_, and t_180_ all *P*s > 0.05).

Blood samples were centrifuged at ∼10,000 *g* for 10 min at 4°C. Plasma was then pipetted off and stored at –20°C for up to 4 weeks before measurement via enzyme immunoassay. All lab animal procedures were approved under the Swarthmore College Institutional Animal Care and Use Committee and met the standards of the Association for Assessment and Accreditation of Laboratory Animal Care.

### Testosterone enzyme immunoassay

Plasma samples were analyzed for testosterone via an enzyme immunoassay kit (K032-H, Arbor Assays, Ann Arbor, MI). Validation of this kit was performed by comparing the slopes of our standard curve and serially diluted pooled degu plasma. Parallelism assumptions were confirmed via ANCOVA (*F*_1,20 _= 1.44, *P* = 0.244). Briefly, we added plasma (up to 128 µL) to 200 µL of phosphate buffer saline (0.1M, pH 7.4), triple-extracted with diethyl ether in a dry ice/methanol bath, and then dried samples under N_2_ gas. Samples were then reconstituted with 125 µL of kit-supplied assay buffer and kept at 4°C overnight. Samples were plated in duplicate and then processed according to the manufacturer's instructions. Samples with estimated concentrations above or below the linear portions of the standard curve (above 4 ng/mL or below 0.102 ng/mL) were re-run with a more appropriate dilution factor. The manufacturer reported an assay sensitivity of 15.2 pg/mL. Samples from different time points were evenly spread across different assay plates; inter-plate CV was 9.6% and intra-plate CV was 3.6%.

### Statistical analyses

To determine whether baseline testosterone levels showed significant diel variation, we ran a linear mixed model with baseline testosterone as the target factor. Baseline testosterone was log-transformed to meet the assumption of normality, as verified via a Shapiro-Wilk test. Time of day (02:00, 06:00, 10:00, 14:00, 18:00, or 22:00) was included as a fixed factor and degu ID nested within the sampling cohort as a random variable, as most individual degus were measured during one daytime period and one nighttime period. To examine more generally whether degus had higher baseline testosterone levels during the night than during the day, we did a paired t-test to compare log-transformed baseline testosterone from each individual degus' daytime and nighttime sampling period. The Benjamini-Hochberg procedure (with a 5% failed discovery rate) was used to control for these multiple comparisons using the same data.

To determine whether GnRH injections significantly increased circulating testosterone levels and whether GnRH-induced testosterone levels varied with time of day, we ran a linear mixed model with testosterone as the target factor, time (10:00 baseline, 10:00 post-GnRH, 14:00 baseline, 14:00 post-GnRH, 18:00 baseline, and 18:00 post-GnRH) as a fixed factor, and degu ID as a random variable.

We ran linear regressions between log-transformed daytime baseline testosterone, log-transformed nighttime baseline testosterone, and daytime post-GnRH testosterone to determine whether levels were significantly correlated with one another. All analyses were run in SPSS (version 27) at an alpha level of 0.05.

## Results

### Diel variation in baseline testosterone levels

**Fig. 3.**
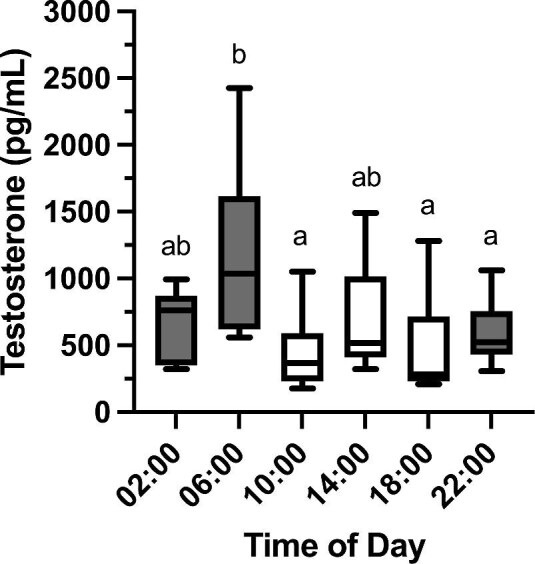
Baseline plasma testosterone concentrations of male degus at six different time points (*n* = 6 per time point). Different letters represent significant differences between time points. Shaded and white boxes represent sampling periods during lights-off and lights-on, respectively. Boxes represent 25th–75th percentiles, middle lines represent medians, and whiskers represent minimum and maximum values.

Baseline testosterone levels showed significant diel variation (Fig. 3; F_5,29.99_ = 2.90, *P* = 0.03, adjusted *a *< 0.05). Baseline testosterone levels at 06:00 were higher than levels early in the dark phase (22:00, post-hoc LSD, *P* = 0.049) but not in the middle of the dark phase (02:00, *P* = 0.102). Levels at 06:00 were also significantly higher than levels early and late in the light phase (10:00, *P* = 0.004; 18:00, *P* = 0.003) but not during the middle of the light phase (14:00, *P* = 0.089). In general, degus had higher baseline testosterone levels during the nighttime than during the daytime (paired *t*-test, t_16_ = 2.48, *P* = 0.024, adjusted *a *< 0.025).

### Lack of diel variation in GnRH-induced testosterone levels

**Fig. 4.**
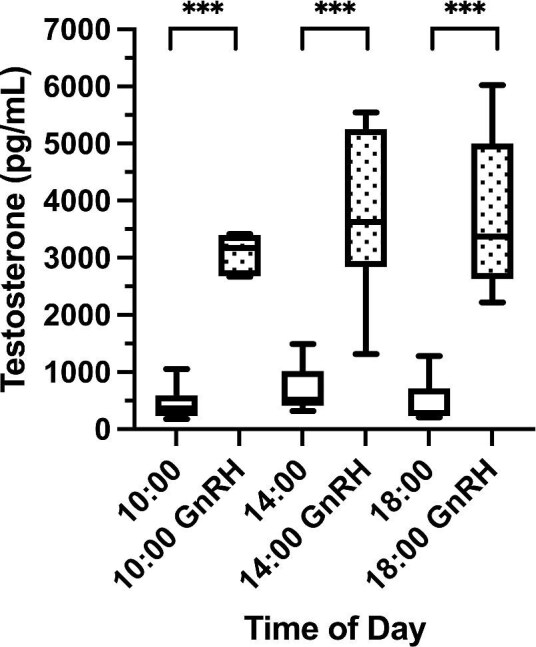
Male degu daytime baseline testosterone levels (white boxes, *n* = 6 per time point) and testosterone levels 2 h after injection of gonadotropin-releasing hormone (GnRH) (stippled boxes, *n* = 6 per time point). *** represent significant differences (*P* < 0.001) between baseline and post-GnRH testosterone levels within a time point. Boxes represent 25th–75th percentiles, middle lines represent medians, and whiskers represent minimum and maximum values.

After injecting degus with GnRH, testosterone levels increased by a factor of 8.8 ± 1.2 (mean ± SEM; F_6,35.7_ = 30.78, *P* < 0.001, post-hoc LSD all *P*s < 0.05 between each baseline and post-GnRH timepoint). Post-GnRH testosterone levels did not significantly vary with time of day, however (Fig. 4; post-hoc LSD all *P*s > 0.05).

### Correlations between daytime, nighttime, and GnRH-induced testosterone levels

**Fig. 5.**
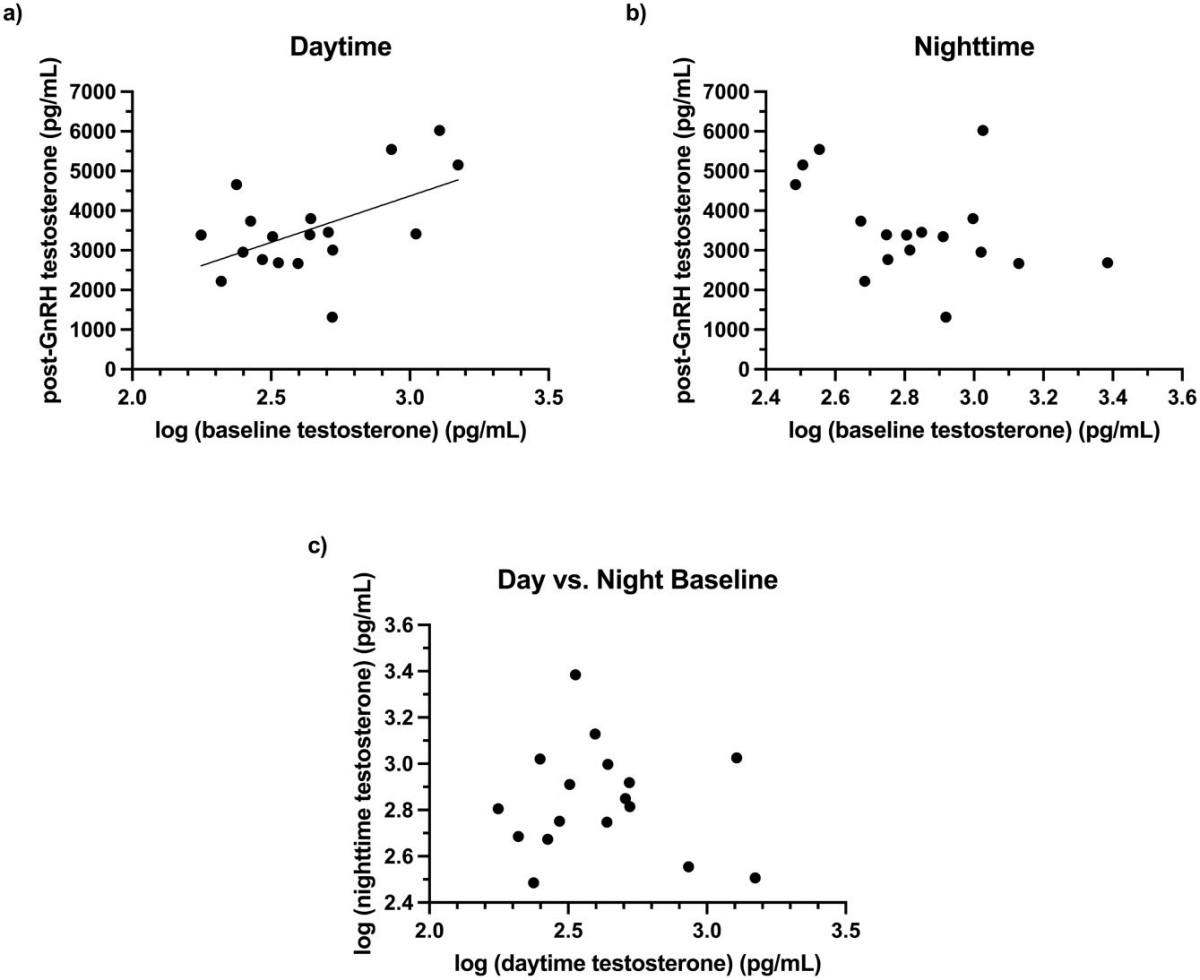
Correlations between (**a)** daytime baseline and daytime post-gonadotropin-releasing hormone (GnRH) testosterone levels (*n* = 18; *r*^2^ = 0.29), (**b)** nighttime baseline and daytime post-GnRH testosterone levels (*n* = 17), and (**c)** daytime and nighttime baseline testosterone levels (*n* = 17) in individual male degus. The equation for the best-fit line in (a) is: *y* = 2340*x*–2648.

While there was a significant positive correlation between daytime baseline testosterone levels and daytime post-GnRH testosterone levels (Fig. 5a; F_1,16_ = 6.48, *P* = 0.02, *r*^2^ = 0.29), there was no significant correlation between nighttime baseline testosterone levels and daytime post-GnRH testosterone levels (Fig. 5b; F_1,15_ = 2.61, *P* = 0.13, *r*^2^ = 0.15). Daytime and nighttime baseline testosterone levels were also not significantly correlated with one another (Fig. 5c; F_1,15_ = 0.13, *P* = 0.72, *r*^2^ = 0.01).

## Discussion

For this study, we measured baseline testosterone levels in laboratory male degus across six time points in a 24-h cycle and gave degus GnRH challenges during lights-on periods. Our hypothesis that baseline testosterone levels peak during the inactive period was supported, as baseline testosterone levels were significantly higher during pooled night timepoints compared to pooled day timepoints. Daytime GnRH injections significantly elevated testosterone levels by several orders of magnitude compared to both daytime and nighttime baseline testosterone levels. However, our prediction that daytime GnRH-induced testosterone levels would be positively related with nighttime testosterone levels was not supported, although we did find a positive relationship between daytime baseline testosterone and GnRH-induced testosterone levels.

Our finding that male degu baseline testosterone levels peak during the inactive period aligns with patterns found in other diurnal mammal species such as the northern palm squirrel (*Funambulus pennantii)* and the rhesus macaque (*Macaca mulatta*), which both display peak testosterone levels during the evening (Perachio et al. 1977; Haldar et al. 2006). Studies in nocturnal mammals have also found that baseline testosterone levels peak during the inactive period, as mouse lemurs (*Microcebus murinus)*, owl monkeys (*Aotus trivirgatus*), and domestic mice (*Mus musculus*) (Dixson and Gardner 1981; Perret 1985; Li et al. 2020) display their highest levels during the daytime. Studies in diurnal birds also align with our results, as great tits (Greives et al. 2021), house sparrows (Needham et al. 2017), and domestic ducks (Balthazart 1976) show peak testosterone levels during the nighttime.

While 06:00 was the only nighttime sampling point where baseline testosterone levels were significantly higher than a daytime sampling point, we found that all pooled nighttime sampling points were significantly higher than all pooled daytime sampling points. Higher testosterone levels during the night could facilitate spermatogenesis, as mammals tend to have higher sperm count levels during their inactive period (Li et al. 2020). The inactive period can be an important time for spermatogenesis, as the optimal temperature for sperm development tends to be a few degrees lower than core mammalian body temperatures (Nakamura et al. 1987), and body temperatures tend to be lower during periods of sleep (Szymusiak 2018). Indeed, degu body temperature is positively related with activity level and is generally lower during the nighttime (Refinetti 1996), which suggests this time period may be optimal for sperm development. Degu testes are likely sensitive to high temperatures, as morphological features help keep epididymides ∼4.8°C lower than core body temperature (Contreras and Rosenmann 1982); future studies should examine whether spermatogenesis activity is higher during the nighttime vs. the daytime in male degus. Peak testosterone levels during the inactive period could also facilitate mating behaviors during the nighttime, as this is when captive female degus are more likely to mate (Mahoney et al. 2011). Information is lacking regarding the time of day that degus breed under wild, free-living conditions, but the determination of their typical mating hours may reflect predawn testosterone peaks.

Testosterone levels in male degus after GnRH challenges were several orders of magnitude higher than peak baseline testosterone levels, which contrasts with Grieves et al. (2021), where peak baseline testosterone levels in great tits (*Parus major*) were equivalent to those stimulated via GnRH challenges. Our findings suggest that degus never reach their maximal testosterone production levels under baseline conditions, although it is possible that degus may be able to upregulate testosterone levels under appropriate stimuli, such as access to fertile females or encounters with unknown males (Gleason et al. 2009). Alternatively, GnRH injections may simply elevate testosterone levels far above natural peak levels in degus. No other studies have performed GnRH challenges in degus, so we cannot compare these testosterone levels with other published data– however, baseline testosterone levels from our study seemed well within the range of those reported in wild degus (Correa et al. 2024b).

We also found that daytime but not nighttime baseline testosterone levels were significantly and positively related with GnRH-induced testosterone levels, which is the opposite of our predictions based on findings in great tits (Greives et al. 2021). We speculate these relationships may be caused by our sampling regimen, as both daytime baseline testosterone and post-GnRH testosterone levels were collected on the same day, whereas nighttime baseline testosterone levels were collected 2 weeks before or after GnRH challenges. This may suggest a lack of repeatability in male degu testosterone measures, yet Needham et al. (2017) found that daytime baseline, nighttime baseline, and post-GnRH challenge testosterone levels were all highly repeatable in captive, photostimulated male house sparrows (*Passer domesticus*). Additionally, Gleason et al. (2012) found that changes in testosterone levels after GnRH injections were repeatable over a 3 week period in captive male California mice (*Peromyscus californicus*). Future studies in degus should examine the repeatability of both baseline and GnRH-induced testosterone levels. A lack of correlation between nighttime baseline testosterone levels and post-GnRH testosterone levels in our study could be due to nonpeak nighttime levels (22:00 and 02:00) obscuring trends- future studies should use a larger sample size to exclusively examine relationships between peak nighttime baseline levels (06:00) with post-GnRH levels.

There is still some uncertainty, however, as to what exactly GnRH-induced testosterone levels represent. GnRH-induced testosterone levels may be supraphysiological and unrealistic, or alternatively, they may represent levels reached under specific stimuli (Wingfield et al. 1990; McGlothlin et al. 2008; Gleason et al. 2009). Future studies should assess whether male degus elevate testosterone levels in response to female presence and/or mate competition, and if these elevations ever reach the same levels as those induced by GnRH injections. Additionally, we did not give GnRH injections during the inactive period, and it is possible that GnRH-induced testosterone levels could vary with time of day. We find this unlikely, however, as diel rhythms in testosterone levels are likely driven by pulsatile release of endogenous GnRH from the hypothalamus (Gore 1998); a GnRH challenge should override that pharmacologically, and should therefore fully stimulate the HPG-axis. Unless there are diel changes in pituitary sensitivity to GnRH or Leydig cell sensitivity to luteinizing hormone, we find it unlikely that testosterone response to GnRH challenges would vary over a 24-h period. Regardless, future studies should assess whether degus and other mammals show diel variation in their testosterone response to GnRH stimulation.

In conclusion, our findings indicate that male degu testosterone levels peak during the inactive period. Sampling male degus during their inactive period would likely provide better resolution for correlations between testosterone levels and reproductive success, although we recognize the logistical challenges of obtaining such samples. Given that we did not find a significant correlation between daytime GnRH-induced testosterone and baseline nighttime testosterone levels, without further validation experiments we doubt that using daytime GnRH challenges could serve as a good proxy for peak baseline testosterone levels. Methodologically, there are tradeoffs between collecting baseline vs. post-GnRH testosterone samples, as the former requires a greater volume of blood, but the latter requires more handling time and transportation of GnRH into the field. Future research is also needed to determine whether GnRH-induced testosterone, itself, could be a good metric for reproductive performance and investment.

## Data Availability

Readers are welcome to contact the corresponding author for access to the data used in this article.
